# Identification of Prion Disease-Related Somatic Mutations in the Prion Protein Gene (*PRNP*) in Cancer Patients

**DOI:** 10.3390/cells9061480

**Published:** 2020-06-17

**Authors:** Yong-Chan Kim, Sae-Young Won, Byung-Hoon Jeong

**Affiliations:** 1 Korea Zoonosis Research Institute, Jeonbuk National University, Iksan, Jeonbuk 54531, Korea; kych@jbnu.ac.kr (Y.-C.K.); gkfh32@jbnu.ac.kr (S.-Y.W.); 2 Department of Bioactive Material Sciences, Jeonbuk National University, Jeonju, Jeonbuk 54896, Korea

**Keywords:** cancer, prion, somatic mutation, aggregation, CJD, FFI, GSS, p53

## Abstract

Prion diseases are caused by misfolded prion protein (PrP^Sc^) and are accompanied by spongiform vacuolation of brain lesions. Approximately three centuries have passed since prion diseases were first discovered around the world; however, the exact role of certain factors affecting the causative agent of prion diseases is still debatable. In recent studies, somatic mutations were assumed to be cause of several diseases. Thus, we postulated that genetically unstable cancer tissue may cause somatic mutations in the prion protein gene (*PRNP*), which could trigger the onset of prion diseases. To identify somatic mutations in the *PRNP* gene in cancer tissues, we analyzed somatic mutations in the *PRNP* gene in cancer patients using the Cancer Genome Atlas (TCGA) database. In addition, to evaluate whether the somatic mutations in the *PRNP* gene in cancer patients had a damaging effect, we performed *in silico* analysis using PolyPhen-2, PANTHER, PROVEAN, and AMYCO. We identified a total of 48 somatic mutations in the *PRNP* gene, including 8 somatic mutations that are known pathogenic mutations of prion diseases. We identified significantly different distributions among the types of cancer, the mutation counts, and the ages of diagnosis between the total cancer patient population and cancer patients carrying somatic mutations in the *PRNP* gene. Strikingly, although invasive breast carcinoma and glioblastoma accounted for a high percentage of the total cancer patient population (9.9% and 5.4%, respectively), somatic mutations in the *PRNP* gene have not been identified in these two cancer types. We suggested the possibility that somatic mutations of the *PRNP* gene in glioblastoma can be masked by a diagnosis of prion disease. In addition, we found four aggregation-prone somatic mutations, these being L125F, E146Q, R151C, and K204N. To the best of our knowledge, this is the first specific analysis of the somatic mutations in the *PRNP* gene in cancer patients.

## 1. Introduction

Prion diseases are fatal and irreversible neurodegenerative diseases caused by a deleterious form of the prion protein (PrP^Sc^) derived from normal prion protein (PrP^C^) [1,2]. These diseases are accompanied by spongiform generation and astrocytosis in brain lesions. Prion diseases are the only zoonotic dementia with such a large host ranges, including scrapie in sheep and goats, bovine spongiform encephalopathy (BSE) in cattle, chronic wasting disease (CWD) in elk and deer, and Creutzfeldt–Jakob disease (CJD), fatal familial insomnia (FFI), and Gerstmann–Sträussler–Scheinker syndrome (GSS) in humans. In CJD, there are three representative types, including sporadic CJD, familial CJD, and iatrogenic CJD [3,4,5,6,7,8,9,10,11,12].

It has been approximately three centuries since scrapie in sheep was first discovered in 1732 [5]. However, the exact roles on certain factors influencing the causative agent of the disease have not been fully explained thus far. However, in recent studies, genetic variations including somatic mutations or postzygotic mutations have been shown to cause several diseases, including neurofibromatosis, McCune–Albright disease, paroxysmal nocturnal hemoglobinuria, incontinentia pigmenti, several types of cancer, and Alzheimer’s disease [13,14,15]. In human prion diseases, point mutations in the prion protein gene (*PRNP*), which encodes PrP, induce familial forms of human prion diseases, including familial CJD, GSS, and FFI [6,16,17]. In addition, spontaneous *de novo* mutations in the *PRNP* gene were detected in several types of prion diseases, including sporadic CJD and GSS [18,19,20,21]. In this regard, somatic mutations in the *PRNP* gene can be assumed to be a cause of prion diseases. In addition, previous studies have been reported that cancer tissues showed more genetic instability and elevated expression of the *PRNP* gene. Thus, we postulated that cancer tissue may induce somatic mutations in the *PRNP* gene, which can trigger the onset of prion diseases [22,23,24,25].

To find somatic mutations in the *PRNP* gene in cancer tissues, we searched the information on somatic mutations in the *PRNP* gene in cancer patients using the Cancer Genome Atlas (TCGA) database [26]. In addition, we compared the information on the somatic mutations in the *PRNP* gene in cancer patients with that of previously reported pathogenic mutations associated with prion diseases and with the total cancer patient population. Furthermore, to assess whether the somatic mutations in the *PRNP* gene in cancer patients are deleterious, we performed *in silico* annotation using PolyPhen-2, PANTHER, PROVEAN, and AMYCO [27,28,29,30,31,32].

## 2. Results

### 2.1. Previously Reported Prion Disease-Related Mutations

Genetic forms of human prion diseases include CJD, FFI, and GSS. Previous pathogenic mutations involved in genetic forms of prion diseases are described in Table 1. In detail, genetic CJD has been reported to carry G114V, D178N-129V, V180I, T183A, T188K, E196K, E196A, E200K, E200G, V203I, R208H, V210I, E211Q, I215V, M232R, and P238S mutations; double octapeptide deletions; and octapeptide insertions in the *PRNP* gene. In addition, FFI has been reported to carry D178N-129M mutations in the *PRNP* gene. Finally, GSS has been reported to carry P102L, P105L, P105T, P105S, A117V, G131V, Y145*, Q160*, V176G, H187R, F198S, D202N, Q212P, Q217R, Y226*, Q227*, and M232T mutations in the *PRNP* gene. Unique phenotypes of dementia have been reported to carry S17G, P39L, Y163*, D167N, D187fs, and R208C mutations in the *PRNP* gene.

### 2.2. 48 Somatic Mutations in the PRNP Gene in Cancer Patients

**Figure 1:**
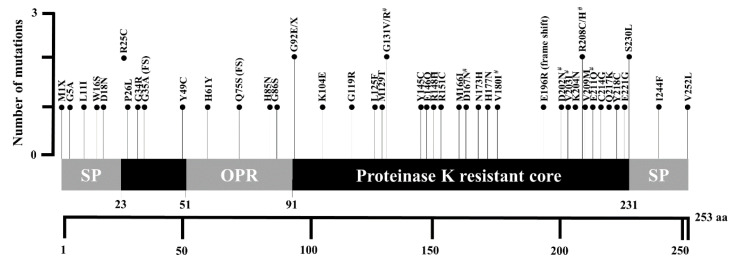
Schematic map of human prion protein (PrP) with somatic mutations of the prion protein gene (*PRNP*) in cancer patients. Sharps indicate previous reported pathogenic mutations of prion diseases. SP: signal peptide; OPR: octapeptide repeat region.

Using cBioPortal, we identified a total of 48 somatic mutations in the *PRNP* gene in 10,953 cancer patients. The detailed information is described in Table 2. Among the 48 mutations, 8 mutations, these being G131V, D167N, V180I, D202N, V203I, R208C, R208H, and E211Q (shaded boxes), were previously reported pathogenic mutations of prion diseases (Table 1). The locations of the identified somatic mutations are visualized in Figure 1. A total of five somatic mutations, these being M1X, G5A, L11I, W16S, and D18N, were located in the N-terminal signal peptide region. A total of five somatic mutations, including R25C, P26L, G34R, and G35A (fs), were located in the region of codons 23–51. A total of four somatic mutations, these being H61Y, Q75S (fs), H85N, and G86S, were located in an octapeptide repeat region. A total of 30 somatic mutations, these being G92E, G92X, K104E, G119R, L125F, M129T, G131V, G131R, Y145C, E146Q, R148H, R151C, M166I, D167N, N173H, H177N, V180I, E196R (FS), D202N, V203I, K204N, R208C, R208H, V209M, E211Q, C214G, Q217K, Y218C, E221G, and S230L, were located in the proteinase K resistant core region. Finally, two somatic mutations, I244F and V252L, were located in the C-terminal signal peptide region. Notably, of the 25 patients for whom we had access to cancer metastasis information, we observed no evidence of distant metastasis in 22 (92%) of them (Table 2).

**Figure 2:**
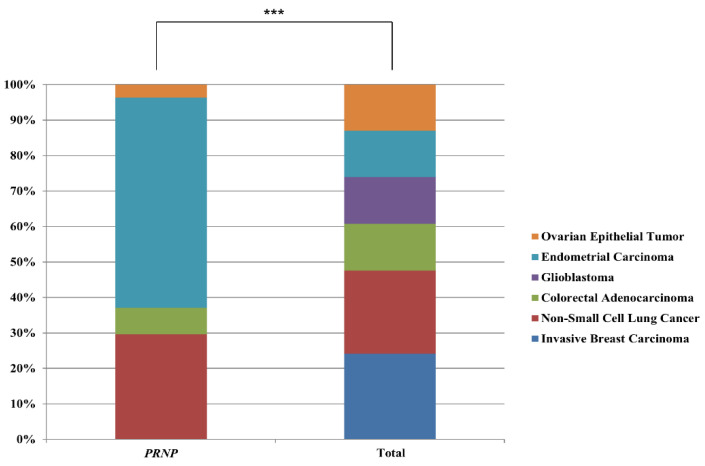
Comparison of distributions of patients harboring somatic mutations of the *PRNP* gene according to six major cancer types. *PRNP*: distribution of cancer patients harboring somatic mutations of the *PRNP* gene in six major cancer types. Total: distribution of cancer patients harboring somatic mutations in six major cancer types. *** indicates *p* < 0.001.

In addition, we compared the information on cancer patients who carried somatic mutations of the *PRNP* gene with that of the total cancer patient population. The detailed information is described in Table 3. In detail, we found 48 *PRNP* somatic mutations carriers (subgroup 1) and 8 *PRNP* pathogenic somatic mutation carriers of prion disease (subgroup 2). Subgroup 1 accounts for 0.43% of the total cancer patient population assessed, while subgroup 2 accounts for 0.07% of the total cancer patient population. Subgroup 2 accounts for 17.02% of subgroup 1. The age of diagnosis in the total cancer patient population was 59.1 ± 14.5 years, in subgroup 1 was 66 ± 12.8 years, and in subgroup 2 was 66.3 ± 7.8 years. Notably, compared to the age of diagnosis in the total cancer patient population, those of subgroups 1 showed significant differences with *p*-values of 0.000742. In addition, there were also significant differences in the mutation count distributions between the total cancer patient population and subgroups 1 and 2, with *p*-values < 0.00001 and 2.3 × 10^−11^, respectively. Specifically, a large number of the total cancer patient population displayed mutation counts <100 (67.3%). By contrast, subgroups 1 and 2 primarily displayed mutation counts >280 (83.3% and 100%, respectively). The distribution of cancer type between the total cancer patient population and subgroup 1 showed statistical significance. (*p* = 6 × 10^−12^). Specifically, the percentage of non-small cell lung cancer patients in subgroup 1 (16.7%) was increased by 1.7-fold compared to the total cancer patient population (9.6%). The percentage of endometrial carcinoma patients in subgroup 1 (33.3%) was increased by 6.3-fold compared to the total cancer patient population (5.3%). The percentage of non-small cell lung cancer patients in subgroup 2 (12.5%) was increased by 1.3-fold compared to the total cancer patient population (9.6%). The percentage of colorectal adenocarcinoma patients in subgroup 2 (12.5%) was increased by 2.3-fold compared to the total cancer patient population (5.4%). The percentage of endometrial carcinoma patients in subgroup 2 (25.0%) was increased by 4.7-fold compared to the total cancer patient population (5.3%). Notably, although invasive breast carcinoma and glioblastoma account for a high percentage of the total cancer patient population (9.9% and 5.4%, respectively), somatic mutations in the *PRNP* gene have not been identified in these two cancer types. Strikingly, distributions of six major cancer types (invasive breast cancer, non-small cell lung cancer, colorectal adenocarcinoma, glioblastoma, endometrial carcinoma, and ovarian epithelial tumor) of the patients with somatic mutations of the *PRNP* gene were shown to be significantly different from distributions of the six major cancer types of total patients (Figure 2).

### 2.3. In Silico Annotation of Mutations in the PRNP Gene

**Figure 3:**
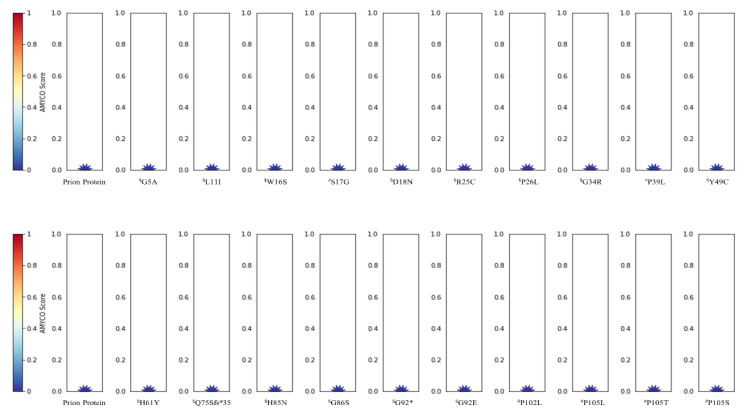

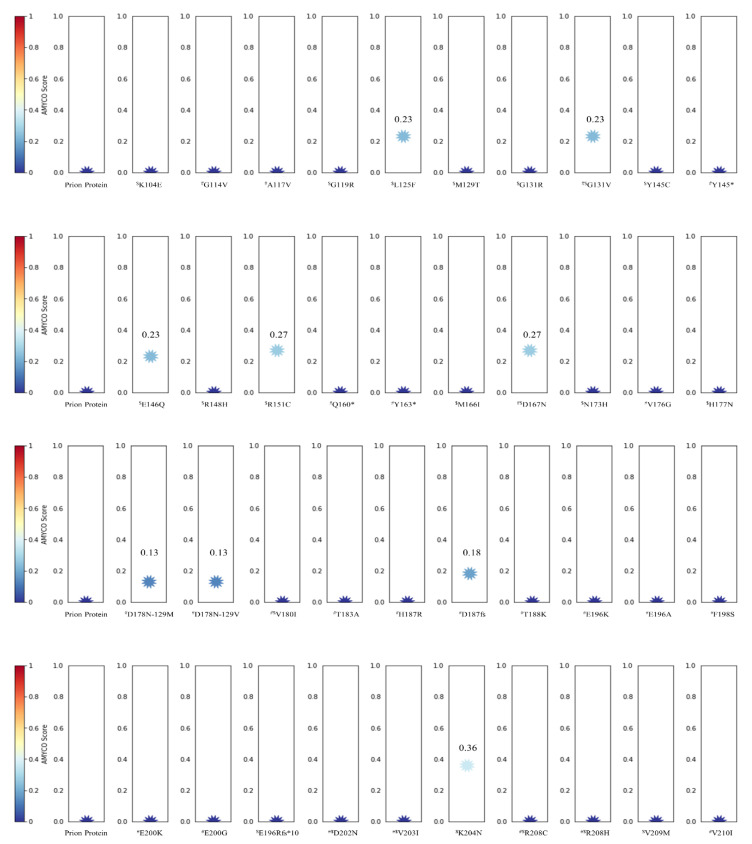

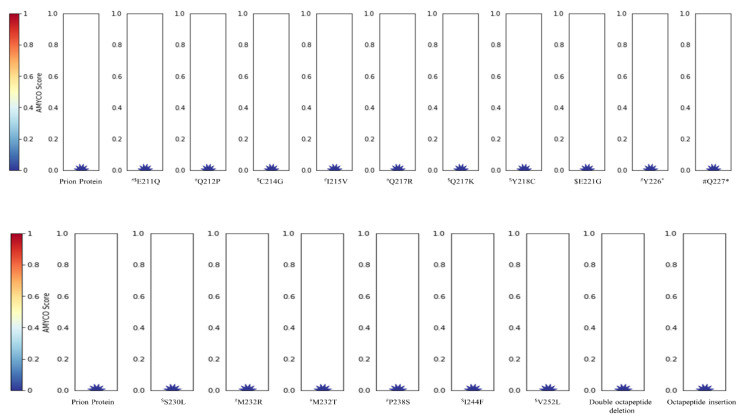
Prediction on amyloid propensity of prion protein (PrP) according to somatic mutations of the prion protein gene (*PRNP*) gene in cancer patients. ^$^ indicates somatic mutations of the *PRNP* gene in cancer patients. ^#^ indicates previously reported pathogenic mutations of the *PRNP* gene in prion diseases.

The impact of somatic mutations in PrP identified in this study and previously reported pathogenic mutations was analyzed by PolyPhen-2, PANTHER, and PROVEAN. The detailed information is described in Table 4. Notably, six somatic mutations, these being P26L, G34R, G119R, G131V, C214G, and Y218C, and five pathogenic mutations, these being P102L, P105L, G114V, H187R, and E200G were predicted to be deleterious by all three programs. We also evaluated the amyloid propensity of the mutations in the *PRNP* gene by AMYCO. The detailed information is described in Figure 3. Interestingly, nine mutations, these being L125F, G131V, E146Q, R151C, D167N, D178N-129M, D178N-129V, D187fs, and K204N, had an increased amyloid propensity with values of 0.23, 0.23, 0.23, 0.27, 0.27, 0.13, 0.13, 0.18, and 0.36, respectively. Among the six somatic mutations of cancer patients, four were newly identified amyloid-prone somatic mutations—L125F, E146Q, R151C, and K204N.

## 3. Discussion

In the present study, we identified a total of 48 somatic mutations in the *PRNP* gene in 10,967 cancer patients. Prion diseases are considered to be monogenic diseases, and the association of the *PRNP* gene with the onset of prion diseases was confirmed by genome-wide association studies (GWAS) [33,34]. Thus, the somatic mutations in the *PRNP* gene in cancer tissues are very important. Of the 48 somatic mutations, 8 were previously confirmed pathogenic mutations of prion diseases. Although we cited pathogenic mutations of the *PRNP* gene from previous studies, the penetrance on the pathogenic mutations of the *PRNP* gene was still debatable. In a previous study, several mutations including V180I, V210I, and M232R were found in healthy controls and these mutations showed low penetrance under 10%. Thus, these results suggest that these mutations were benign variants or low-risk variants [35]. In the present study, we further analyzed previously reported pathogenic mutations using *in silico* programs. Notably, V180I, V210I, and M232R mutations were predicted to be low amyloid propensity and benign by some programs (Figure 3, Table 4). Further study to confirm the deleterious effects of the mutations is needed in the future. In addition, cancer patients carrying these somatic mutations showed no evidence of distant metastasis (Table 2). Since PrP contributes to metastasis in several types of cancers, somatic mutations of the *PRNP* gene, which can affect normal function of PrP, may impact the property of distant metastasis [36,37]. Further validation of distant metastatic property in cancer cells carrying *PRNP* mutations is highly desirable in the future. Interestingly, the age of patients carrying somatic mutations (66 ± 12.8 years) was much older than that of the total cancer patient population (59.1 ± 14.5 years) (Table 2). Since sporadic prion disease has been reported to develop around the ages of 60–70, this was a very interesting finding [38]. In addition, since PrP contributes to several properties of cancer including tumorigenesis, protection of apoptotic stress, and metastasis, further study of the relationship between age and several characteristics of cancers mediated by somatic mutations of the *PRNP* gene is needed in the future [36,37,39,40]. Furthermore, 83.3% of patients carrying somatic mutations of the *PRNP* gene showed over 280 mutation counts. This distribution is significantly different from that of the total cancer patient population. In addition, the cancer type of the patients carrying somatic mutations was also significantly different from the total cancer patient population. Notably, although invasive breast carcinoma (9.9%) and glioblastoma (5.4%) account for high percentage of the total cancer patient population, somatic mutation of the *PRNP* gene has not been identified in these two cancer types. Specifically, because somatic mutations of the *PRNP* gene in glioblastoma, which occurs in the brain, can be masked by a diagnosis of dementia, the absence of somatic mutations in the *PRNP* gene in glioblastoma can be explained. Previous study has been reported that transgenic mice carrying a pathogenic mutation of the *PRNP* gene can lead to spontaneous prion disease [41,42]. In addition, a recent study has been reported that PrP^Sc^ were also detected in healthy people, who have not been diagnosed with prion diseases [43]. On the basis of these studies, we postulated that cancer patients carrying pathogenic somatic mutation of the *PRNP* gene may produce a basal level of PrP^Sc^ in peripheral tissues and may not be diagnosed with prion disease. However, to date, there is no clinical evidence for this hypothesis. Since PrP protects cancer cells against apoptotic stress and contributes to tumorigenesis, the difference on distribution of somatic mutation of the *PRNP* gene may be induced depending on the degree of contribution of PrP to different cancer types [39,40]. Further study to verify functional role of prion protein in various cancer types is needed in the future.

We also analyzed somatic mutations of the *PRNP* gene in cancer patients by PolyPhen-2, PANTHER, and PROVEAN. Notably, six somatic mutations—P26L, G34R, G119R, G131V, C214G, and Y218C—and five pathogenic mutations—P102L, P105L, G114V, H187R, and E200G—were considered to be damaging to PrP function by all three programs. Since PrP^C^ has also been reported to be related to the survival signaling pathway and copper ion-associated enzyme activity, these somatic mutations may impact the normal function of PrP [44]. In addition, we predicted the amyloid propensity of the somatic mutations in the *PRNP* gene in cancer patients. Notably, four somatic mutations—L125F, E146Q, R151C, and K204N—were newly identified amyloid-prone somatic mutations in the *PRNP* gene in cancer patients. In addition, we found several nonsense somatic mutations. These nonsense mutations induced PrP cleavage, which can affect the survival signaling pathway or the aggregation of PrP [6,17,45]. Thus, further investigation of the mutation counts, the absence of somatic mutations in glioblastoma, and these nonsense mutations are highly desirable in the future.

In recent cancer studies, p53 has been demonstrated to play a pivotal role in several types of cancer and showed prion-like aggregation. Aggregation of p53 causes additional deleterious effects in cancer patients [46,47,48]. In addition, although cross-seeding activity between aggregated p53 and PrP^Sc^ has not been investigated thus far, the similarity of cross-seeding activities in other misfolded proteins suggests that aggregated p53 may act as a seed to induce the aggregation of PrP [49]. Thus, the detection of PrP^Sc^ in cancer patients using Western blot or protein misfolded cyclic amplification (PMCA) is highly desirable in the future.

## 4. Material and Methods

### 4.1. Information on the Somatic Mutations in the PRNP Gene in Cancer Patients

The protein sequence of human PrP in Figure 1 was obtained from GenBank at the National Center for Biotechnology Information (NCBI, AAH22532.1). Previously reported pathogenic mutations associated with prion disease were collected from previous studies [6,16,17]. The data on somatic mutations of the *PRNP* gene in cancer patients were extracted from TCGA database using cBioPortal (http://www.cbioportal.org/) [26]. Among total mutations of the *PRNP* gene, germline mutations were excluded using the filtering option. Somatic mutations were analyzed using reference sequence from GenBank at the NCBI (NM_000311.5). Clinical data including somatic mutations, age of diagnosis, sex, mutation count, cancer type, and allele frequencies were retrieved from cBioPortal and the data were read by R (https://www.r-project.org/).

### 4.2. Statistical Analysis

Statistical analyses were performed using SAS version 9.4 (SAS Institute Inc., Cary, NC, USA). The differences in the distributions of sex, mutation count, and cancer type between the total cancer patient population and the subgroups were compared using the χ^2^ test. The age of diagnosis was reported as the mean values ± standard deviation (SD). Statistical significance based on *p*-values was calculated with a one-tailed Student’s *t*-test for single comparison.

### 4.3. In Silico Evaluation of Somatic Mutations in the PRNP Gene in Cancer Patients

Somatic mutations in the *PRNP* gene in cancer patients were evaluated by PolyPhen-2 (http://genetics.bwh.harvard.edu/pph2/), PANTHER (http://www.pantherdb.org/), PROVEAN (http://provean.jcvi.org/index.php), and AMYCO (http://bioinf.uab.es/amycov04/) [27,28,29,30]. The effects of sequence variation were evaluated on the basis of protein structure by PolyPhen-2. The PolyPhen-2 score corresponds to the substitution effect and ranges from 0.0 to 1.0. The prediction outcome can be presented as “Benign”, “Possibly damaging”, or “Probably damaging” on the basis of the score. PROVEAN assessed amino acid substitution by building up and comparing the clusters of related sequences and calculating the score. Scores below −2.5 are predicted as “Deleterious” and scores above −2.5 are predicted as “Neutral”. PANTHER analyzes data in groups on the basis of similarities in molecular function and biological processes. PANTHER analyzed preservation time to estimate the substitution of amino acids. The interpretation of preservation time is described as follows: “Probably damaging” is greater than 450; “Possibly damaging” is between 200 and 450; “Probably benign” is less than 200. AMYCO evaluates the impact of variations on the aggregation propensity of prion-like proteins. The AMYCO score corresponds to the substitution effect and ranges from 0.0 to 1.0.

## 5. Conclusions

In conclusion, we identified 48 somatic mutations in the *PRNP* gene in 10,967 cancer patients. Among them, eight somatic mutations are pathogenic for prion diseases. We identified significantly different distributions of cancer type, mutation count, and the age of diagnosis between the total cancer patient population and cancer patients carrying *PRNP* somatic mutations. We found six somatic mutations, these being P26L, G34R, G119R, G131V, C214G, and Y218C, and five pathogenic mutations, these being P102L, P105L, G114V, H187R, and E200G, which can affect PrP^C^ function, along with four aggregation-prone somatic mutations—L125F, E146Q, R151C, and K204N. To the best of our knowledge, this is the first specific analysis on the somatic mutations of the *PRNP* gene in cancer patients.

## Figures and Tables

**Table 1:** Classification of pathogenic mutations of prion disease according to clinical phenotypes in previous studies.

Clinical Phenotypes	Mutations
Creutzfeldt–Jakob Disease (CJD)	G114V, D178N-129V, V180I, T183A, T188K, E196K, E196A, E200K, E200G, V203I, R208H, V210I, E211Q, I215V, M232R, P238S, double octapeptide deletion, and octapeptide insertions
Fatal familial insomnia (FFI)	D178N-129M
Gerstmann–Sträussler–Scheinker syndrome (GSS)	P102L, P105L, P105T, P105S, A117V, G131V, Y145*, Q160*, V176G, H187R, F198S, D202N, Q212P, Q217R, Y226*, Q227*, and M232T
Others	S17G, P39L, Y163*, D167N, D187fs, and R208C

Others: includes unique phenotypes of dementia. *: stop codon. fs: frame shift.

**Table 2:** Detailed information on the 48 somatic mutations in cancer patients.

Sample ID	Sex	Age	Cancer Type	AJCCMSC	AJCCTSC	Mutation	Mutation Type	Allele Frequency	# Mut
TCGA-4Z-AA7Q-01	M	79	Bladder urothelial carcinoma	M0	T3A	E146Q	Missense	0.19	222
TCGA-5N-A9KM-01	F	73	Bladder urothelial carcinoma	MX	T4A	M1?	Nonstart	0.36	189
TCGA-IR-A3LK-01	F	69	Cervical squamous cell carcinoma	M0	T1B2	G131V	Missense	0.14	1189
TCGA-KN-8428-01	M	71	Chromophobe renal cell carcinoma	NA	T2	V203I	Missense	0.20	673
TCGA-CK-5916-01	F	71	Colon adenocarcinoma	M0	T1	V180I	Missense	0.25	1407
TCGA-EE-A29V-06	M	85	Cutaneous melanoma	M0	T3B	G92E	Missense	0.42	1012
TCGA-EB-A41A-01	M	90	Cutaneous melanoma	M0	T4B	L125F	Missense	0.44	1533
TCGA-D3-A2JC-06	F	53	Cutaneous melanoma	M0	T0	M166I	Missense	0.11	3148
TCGA-D3-A8GM-06	M	73	Cutaneous melanoma	M0	T3B	G86S	Missense	0.09	3288
TCGA-YD-A9TA-06	M	75	Cutaneous melanoma	NA	NA	H61Y	Missense	0.29	2782
TCGA-L5-A43J-01	M	90	Esophageal squamous cell carcinoma	MX	T3	R25C	Missense	0.20	700
TCGA-F7-A624-01	M	73	Head and neck squamous cell carcinoma	M0	T2	R208H	Missense	0.16	2744
TCGA-VQ-A91D-01	M	70	Intestinal type stomach adenocarcinoma	M0	T4B	R208C	Missense	0.25	2140
TCGA-67-3771-01	F	77	Lung adenocarcinoma	M0	T1	N173H	Missense	0.12	951
TCGA-44-5644-01	F	51	Lung adenocarcinoma	NA	T2A	G5A	Missense	0.34	908
TCGA-69-A59K-01	F	60	Lung adenocarcinoma	M0	T3	G119R	Missense	0.21	438
TCGA-17-Z023-01	NA	NA	Lung adenocarcinoma	NA	NA	Y49C	Missense	0.23	362
TCGA-17-Z026-01	NA	NA	Lung adenocarcinoma	NA	NA	E211Q	Missense	0.19	873
TCGA-22-5489-01	M	64	Lung squamous cell carcinoma	M0	T1B	Y145C	Missense	0.21	227
TCGA-63-A5MM-01	F	69	Lung squamous cell carcinoma	M0	T2	H177N	Missense	0.23	1070
TCGA-NC-A5HH-01	M	53	Lung squamous cell carcinoma	M0	T1	W16S	Missense	0.27	314
TCGA-G7-6790-01	M	57	Papillary renal cell carcinoma	MX	T1A	Q217K	Missense	0.09	71
TCGA-F9-A97G-01	M	79	Papillary renal cell carcinoma	M0	T3	V252L	Missense	0.20	62
TCGA-AG-A002-01	M	35	Rectal adenocarcinoma	M0	T2	P26L	Missense	0.45	11,438
TCGA-B0-5713-01	F	75	Renal clear cell carcinoma	M0	T3B	E221G	Missense	0.30	97
TCGA-25-1313-01	F	62	Serous ovarian cancer	NA	NA	G131R	Missense	0.37	179
TCGA-VQ-A8PO-01	M	74	Signet ring cell carcinoma of the stomach	M0	T4A	M129T	Missense	0.23	751
TCGA-BR-6452-01	F	78	Stomach adenocarcinoma	M0	T3	I244F	Missense	0.19	5050
TCGA-CG-5717-01	M	58	Stomach adenocarcinoma	M0	T2B	R151C	Missense	0.34	117
TCGA-CG-5723-01	M	83	Stomach adenocarcinoma	M0	T2	V209M	Missense	0.08	1606
TCGA-VQ-A8PP-01	M	76	Tubular stomach adenocarcinoma	M0	T4	K104E	Missense	0.36	1328
TCGA-DX-AB2Z-01	F	87	Undifferentiated pleomorphic sarcoma	NA	NA	R148H	Missense	0.11	69
TCGA-N7-A4Y0-01	F	65	Uterine carcinosarcoma	NA	NA	C214G	Missense	0.64	709
TCGA-ND-A4WC-01	NA	NA	Uterine carcinosarcoma	NA	NA	H85N	Missense	0.20	3669
TCGA-B5-A0JY-01	F	50	Uterine endometrioid carcinoma	NA	NA	K204N	Missense	0.34	9713
TCGA-D1-A17Q-01	F	54	Uterine endometrioid carcinoma	NA	NA	D167N	Missense	0.40	5945
TCGA-AP-A1DV-01	F	59	Uterine endometrioid carcinoma	NA	NA	R25C	Missense	0.52	12,071
TCGA-FI-A2D5-01	F	56	Uterine endometrioid carcinoma	NA	NA	D202N	Missense	0.38	13,874
TCGA-AJ-A3EL-01	F	47	Uterine endometrioid carcinoma	NA	NA	L11I	Missense	0.40	7391
TCGA-AP-A0LT-01	F	57	Uterine endometrioid carcinoma	NA	NA	E196Rfs*10	Frame shift del	0.28	638
TCGA-AP-A1DV-01	F	59	Uterine endometrioid carcinoma	NA	NA	Y218C	Missense	0.42	12,071
TCGA-AX-A3FT-01	F	64	Uterine endometrioid carcinoma	NA	NA	Q75Sfs*35	Frame shift del	0.23	1281
TCGA-B5-A1MX-01	F	47	Uterine endometrioid carcinoma	NA	NA	G92*	Nonsense	0.33	5699
TCGA-BG-A222-01	F	49	Uterine endometrioid carcinoma	NA	NA	G34R	Missense	0.45	4276
TCGA-DF-A2KU-01	F	NA	Uterine endometrioid carcinoma	NA	NA	S230L	Missense	0.30	10,058
TCGA-EO-A22R-	F	56	Uterine endometrioid carcinoma	NA	NA	S230L	Missense	0.30	12,783
TCGA-EY-A1GK-01	F	74	Uterine endometrioid carcinoma	NA	NA	G35Afs*75	Frame shift del	0.30	841
TCGA-A5-A0G2-01	F	57	Uterine serous carcinoma	NA	NA	D18N	Missense	0.18	25,730

AJCCMSC: American Joint Committee on Cancer Metastasis Stage Code. AJCCTSC: American Joint Committee on Cancer Tumor Stage Code. # Mut: Total number of non-synonymous mutation in the sample. M0: No evidence of distant metastasis. MX: Distant metastasis cannot be assessed. NA: Not available. Shaded boxes indicate pathogenic somatic mutations of prion diseases. *: stop codon.

**Table 3:** Summary of information on the cancer patients carrying somatic mutations in the *PRNP* gene.

Characteristics		All Cancer Patients	Subgroup 1	Subgroup 2
Number of patients		10,953	47	8
	% compared to all cancer patients	-	0.43%	0.07%
	% compared to subgroup 1	-	-	17.02%
Number of samples		10,967	48	8
	% compared to all cancer patients		0.44%	0.07%
	% compared to subgroup 1	-	-	16.67%
Diagnosis age (mean ± SD)		59.1 ± 14.5	66 ± 12.8	66.3 ± 7.8
	*p*-value compared to all cancer patients		**0.000742**	0.093018
	*p*-value compared to subgroup 1	-	**-**	0.477389
Sex, *N* (%)	Male	4866 (44.4%)	18 (37.5%)	3 (37.5%)
	Female	5315 (48.5%)	27 (56.2%)	4 (50.0%)
	NA	772 (7.0%)	3 (6.3%)	1 (12.5%)
	Total	10,953	48	8
	*p*-value compared to all cancer patients	-	0.30	0.79
	*p*-value compared to subgroup 1	-	-	0.89
Mutation count	<100	6798 (67.3%)	4 (8.3%)	0 (0%)
	100 ≤ x < 200	1516 (15.0%)	2 (4.2%)	0 (0%)
	200 ≤ x < 280	455 (4.5%)	2 (4.2%)	0 (0%)
	>280	1328 (13.2%)	40 (83.3%)	8 (100%)
	Total	10,097 (100%)	48	8
	*p*-value compared to all cancer patients	-	**<0.00001**	**2.3 × 10^−11^**
	*p*-value compared to subgroup 1	-	-	0.67
Cancer type, *N* (%)	Invasive breast carcinoma	1084 (9.9%)	0 (0%)	0 (0%)
	Non-small cell lung cancer	1053 (9.6%)	8 (16.7%)	1 (12.5%)
	Colorectal adenocarcinoma	594 (5.4%)	2 (4.2%)	1 (12.5%)
	Glioblastoma	592 (5.4%)	0 (0%)	0 (0%)
	Endometrial carcinoma	586 (5.3%)	16 (33.3%)	2 (25.0%)
	Ovarian epithelial tumor	585 (5.3%)	1 (2.1%)	0
	Head and neck squamous cell carcinoma	523 (4.8%)	1 (2.1%)	1 (12.5%)
	Esophagogastric adenocarcinoma	514 (4.7%)	5 (10.4%)	1 (12.5%)
	Diffuse glioma	513 (4.7%)	0 (0%)	0 (0%)
	Renal clear cell carcinoma	512 (4.7%)	1 (2.1%)	0 (0%)
	Well-differentiated thyroid	500 (4.6%)	0 (0%)	0 (0%)
	Prostate adenocarcinoma	494 (4.5%)	0 (0%)	0 (0%)
	Melanoma	448 (4.1%)	5 (10.4%)	0 (0%)
	Bladder urothelial carcinoma	411 (3.7%)	2 (4.2%)	0 (0%)
	Hepatocellular carcinoma	369 (3.4%)	0 (0%)	0 (0%)
	Renal non-clear cell carcinoma	348 (3.2%)	3 (6.3%)	1 (12.5%)
	Sarcoma	255 (2.3%)	1 (2.1%)	0 (0%)
	Cervical squamous cell carcinoma	251 (2.3%)	1 (2.1%)	1 (12.5%)
	Leukemia	200 (1.8%)	0 (0%)	0 (0%)
	Pancreatic adenocarcinoma	184 (1.7%)	0 (0%)	0 (0%)
	Pheochromocytoma	147 (1.3%)	0 (0%)	0 (0%)
	Thymic epithelial tumor	123 (1.1%)	0 (0%)	0 (0%)
	Esophageal squamous cell carcinoma	95 (0.9%)	1 (2.1%)	0 (0%)
	Adrenocortical carcinoma	92 (0.8%)	0 (0%)	0 (0%)
	Pleural mesothelioma	87 (0.8%)	0 (0%)	0 (0%)
	Non-seminomatous germ cell tumor	86 (0.8%)	0 (0%)	0 (0%)
	Ocular melanoma	80 (0.7%)	0 (0%)	0 (0%)
	Seminoma	63 (0.6%)	0 (0%)	0 (0%)
	Mature B-cell neoplasms	48 (0.4%)	0 (0%)	0 (0%)
	Cervical adenocarcinoma	46 (0.4%)	0 (0%)	0 (0%)
	Cholangiocarcinoma	36 (0.3%)	0 (0%)	0 (0%)
	Miscellaneous neuroepithelial tumor	31 (0.3%)	0 (0%)	0 (0%)
	Undifferentiated stomach adenocarcinoma	13 (0.1%)	1 (2.1%)	0 (0%)
	Fibrolamellar carcinoma	3 (<0.1%)	0 (0%)	0 (0%)
	Encapsulated glioma	1 (<0.1%)	0 (0%)	0 (0%)
	Total	10,967	48	8
	*p*-value compared to all cancer patients	-	**6 × 10^−12^**	0.96
	*p*-value compared to subgroup 1	-	-	NA

Subgroup 1: carriers of *PRNP* somatic mutations. Subgroup 2: carriers of *PRNP* pathogenic somatic mutations of prion disease. NA: not available. Shaded boxes indicate pathogenic somatic mutations of prion disease. Bold text indicates statistical significance (*p* < 0.05).

**Table 4:** *In silico* annotations of *PRNP* somatic mutations in cancer patients.

Mutations	PolyPhen-2		PANTHER		PROVEAN	
	Score	Prediction	Preservation Time	Prediction	Score	Prediction
^$^ G5A	0.729	Possibly damaging	176	Probably benign	−1.238	Neutral
^$^ L11I	0.161	Benign	176	Probably benign	−0.306	Neutral
^$^ W16S	0.546	Possibly damaging	176	Probably benign	−1.237	Neutral
^#^ S17G	0.528	Possibly damaging	176	Probably benign	−0.239	Neutral
^$^ D18N	1.000	Probably damaging	176	Probably benign	−0.104	Neutral
^$^ R25C	1.000	Probably damaging	176	Probably benign	−1.801	Neutral
^$^ P26L	0.995	Probably damaging	324	Possibly damaging	−3.263	Deleterious
^$^ G34R	1.000	Probably damaging	220	Possibly damaging	−3.889	Deleterious
^#^ P39L	1.000	Probably damaging	361	Possibly damaging	−4.000	Deleterious
^$^ Y49C	1.000	Probably damaging	176	Probably benign	−2.222	Neutral
^$^ H61Y	0.975	Probably damaging	176	Probably benign	−1.021	Neutral
^$^ H85N	0.975	Probably damaging	176	Probably benign	−0.833	Neutral
^$^ G86S	1.000	Probably damaging	324	Possibly damaging	−0.995	Neutral
^$^ G92E	1.000	Probably damaging	176	Probably benign	−1.253	Neutral
^#^ P102L	1.000	Probably damaging	361	Possibly damaging	−3.392	Deleterious
^#^ P105L	1.000	Probably damaging	324	Possibly damaging	−3.271	Deleterious
^#^ P105T	0.998	Probably damaging	324	Possibly damaging	−2.333	Neutral
^#^ P105S	0.997	Probably damaging	324	Possibly damaging	−1.496	Neutral
^$^ K104E	0.974	Probably damaging	324	Possibly damaging	−1.208	Neutral
^#^ G114V	1.000	Probably damaging	220	Possibly damaging	−2.540	Deleterious
^#^ A117V	0.999	Probably damaging	176	Probably benign	−1.263	Neutral
^$^ G119R	1.000	Probably damaging	361	Possibly damaging	−2.586	Deleterious
^$^ L125F	1.000	Probably damaging	324	Possibly damaging	−0.398	Neutral
^$^ M129T	0.181	Benign	324	Possibly damaging	−1.156	Neutral
^$^ G131R	1.000	Probably damaging	361	Possibly damaging	−2.451	Neutral
^#,$^ G131V	1.000	Probably damaging	361	Possibly damaging	−2.879	Deleterious
^$^ Y145C	0.997	Probably damaging	220	Possibly damaging	−1.742	Neutral
^$^ E146Q	0.992	Probably damaging	361	Possibly damaging	−0.985	Neutral
^$^ R148H	1.000	Probably damaging	361	Possibly damaging	−1.735	Neutral
^$^ R151C	0.009	Benign	220	Possibly damaging	−2.016	Neutral
^$^ M166I	0.000	Benign	30	Probably benign	0.254	Neutral
^#,$^ D167N	0.001	Benign	220	Possibly damaging	−0.631	Neutral
^$^ N173H	0.952	Probably damaging	176	Probably benign	−1.573	Neutral
^#^ V176G	0.998	Probably damaging	361	Possibly damaging	−2.253	Neutral
^$^ H177N	0.313	Benign	220	Possibly damaging	−0.455	Neutral
^#^ D178N-129M	1.000	Probably damaging	361	Possibly damaging	−1.531	Neutral
^#^ D178N-129V	1.000	Probably damaging	361	Possibly damaging	−1.451	Neutral
^#,$^ V180I	0.009	Benign	220	Possibly damaging	−0.11	Neutral
^#^ T183A	0.978	Probably damaging	324	Possibly damaging	−1.785	Neutral
^#^ H187R	0.989	Probably damaging	220	Possibly damaging	−2.607	Deleterious
^#^ T188K	0.996	Probably damaging	324	Possibly damaging	−0.550	Neutral
^#^ E196K	0.624	Possibly damaging	220	Possibly damaging	−0.641	Neutral
^#^ E196A	0.472	Possibly damaging	220	Possibly damaging	−1.206	Neutral
^#^ F198S	0.994	Probably damaging	220	Possibly damaging	−0.792	Neutral
^#^ E200K	0.995	Probably damaging	361	Possibly damaging	−1.478	Neutral
^#^ E200G	0.994	Probably damaging	361	Possibly damaging	−3.245	Deleterious
^#,$^ D202N	1.000	Probably damaging	220	Possibly damaging	−1.118	Neutral
^#,$^ V203I	0.001	Benign	176	Probably benign	−0.004	Neutral
^$^ K204N	0.898	Possibly damaging	220	Possibly damaging	−1.815	Neutral
^#,$^ R208C	1.000	Probably damaging	220	Possibly damaging	−2.324	Neutral
^#,$^ R208H	0.999	Probably damaging	220	Possibly damaging	−0.855	Neutral
^$^ V209M	0.613	Possibly damaging	324	Possibly damaging	−1.03	Neutral
^#^ V210I	0.803	Possibly damaging	220	Possibly damaging	0.039	Neutral
^#,$^ E211Q	0.992	Probably damaging	220	Possibly damaging	−0.36	Neutral
^#^ Q212P	0.930	Possibly damaging	220	Possibly damaging	−1.665	Neutral
^$^ C214G	0.975	Probably damaging	361	Possibly damaging	−4.402	Deleterious
^#^ I215V	0.000	Benign	220	Possibly damaging	−0.099	Neutral
^#^ Q217R	0.961	Probably damaging	220	Possibly damaging	−1.306	Neutral
^$^ Q217K	0.942	Probably damaging	220	Possibly damaging	−1.186	Neutral
^$^ Y218C	1.000	Probably damaging	361	Possibly damaging	−3.626	Deleterious
^$^ E221G	0.651	Possibly damaging	220	Possibly damaging	−1.244	Neutral
^$^ S230L	0.947	Possibly damaging	97	Probably benign	−1.372	Neutral
^#^ M232R	0.082	Benign	91	Probably benign	−1.167	Neutral
^#^ M232T	0.000	Benign	91	Probably benign	−0.825	Neutral
^#^ P238S	1.000	Probably damaging	361	Possibly damaging	−1.189	Neutral
^$^ I244F	0.001	Benign	176	Probably benign	−0.939	Neutral
^$^ V252L	0.990	Probably damaging	176	Probably benign	−0.411	Neutral

^$^ indicates somatic mutations of the *PRNP* gene in cancer patients. ^#^ indicates previously reported pathogenic mutations of the *PRNP* gene in prion diseases. Shaded boxes indicate the mutations that were predicted to be deleterious by all three programs.

## Abbreviations

PrP^Sc^	Misfolded prion protein
PrP^C^	Normal prion protein
PrP	Prion protein
TCGA	The Cancer Genome Atlas
BSE	Bovine spongiform encephalopathy
CJD	Creutzfeldt–Jakob disease
CWD	Chronic wasting disease
FFI	Fatal familial insomnia
GSS	Gerstmann–Sträussler–Scheinker syndrome
PRNP	Prion protein gene
